# Transcriptomics Insights into Phosphorus Stress Response of *Myriophyllum aquaticum*

**DOI:** 10.3390/ijms24054874

**Published:** 2023-03-02

**Authors:** Cancan Jiang, Shengjun Xu, Rui Wang, Qian Sun, Jialiang Zuo, Xuliang Zhuang

**Affiliations:** 1 Key Laboratory of Environmental Biotechnology, Research Center for Eco-Environmental Sciences, Chinese Academy of Sciences, Beijing 100085, China; 2 College of Resources and Environment, University of Chinese Academy of Sciences, Beijing 100049, China; 3 Yangtze River Delta Research Center for Eco-Environmental Sciences, Yiwu 322000, China; 4 Institute of Tibetan Plateau Research, Chinese Academy of Sciences, Beijing 100101, China

**Keywords:** *Myriophyllum aquaticum*, phosphorus stress, transcriptomics, differentially expressed genes, phosphorus metabolism

## Abstract

Through excellent absorption and transformation, the macrophyte *Myriophyllum* (*M.*) *aquaticum* can considerably remove phosphorus from wastewater. The results of changes in growth rate, chlorophyll content, and roots number and length showed that *M. aquaticum* could cope better with high phosphorus stress compared with low phosphorus stress. Transcriptome and differentially expressed genes (DEGs) analyses revealed that, when exposed to phosphorus stresses at various concentrations, the roots were more active than the leaves, with more DEGs regulated. *M. aquaticum* also showed different gene expression and pathway regulatory patterns when exposed to low phosphorus and high phosphorus stresses. *M. aquaticum*’s capacity to cope with phosphorus stress was maybe due to its improved ability to regulate metabolic pathways such as photosynthesis, oxidative stress reduction, phosphorus metabolism, signal transduction, secondary metabolites biosynthesis, and energy metabolism. In general, *M. aquaticum* has a complex and interconnected regulatory network that deals efficiently with phosphorus stress to varying degrees. This is the first time that the mechanisms of *M. aquaticum* in sustaining phosphorus stress have been fully examined at the transcriptome level using high-throughput sequencing analysis, which may indicate the direction of follow-up research and have some guiding value for its future applications.

## 1. Introduction

Large amounts of phosphorus are discharged into natural water due to excessive use and improper management, such as overuse of phosphorous chemical pesticides and fertilizers and reckless mining of phosphate rock, etc., which can lead to a series of environmental problems, including eutrophication, blooming water bodies, and biodiversity reduction in aquatic ecosystems, which also poses a great threat to human health and ecological security [1]. Among the methods for removing phosphorus pollution, biological technologies, such as constructed wetlands (CWs), are efficient and low-cost technologies, and are widely used for phosphorus from wastewater discharged from industry, agriculture, and aquaculture [2,3]. The mechanism of phosphorus removal in constructed wetlands includes substrate adsorption, plant absorption and utilization, and transformation into new sediments and soils, in which plants play an indispensable role [4,5]. It has been reported that aquatic plants have different removal efficiencies in CWs. For example, Phragmites communis and Acorus calamus can fix about 18 kg/hm^2^ and 15 kg/hm^2^ of phosphorus per year, respectively, while cattails can absorb up to 400 kg/hm^2^ of phosphorus per year [6,7]. Therefore, the selection of suitable wetland plants is very important for efficient phosphorus removal.

In recent years, *Myriophyllum spicatum* has been increasingly used in CWs due to its excellent pollutant removal ability, rapid accumulation of biomass, and strong adaptability [8,9,10]. A large number of studies have shown that the removal efficiency of phosphorus for CWs planted with *M. aquaticum* can reach more than 90% [4,11,12]. *M. aquaticum* can not only absorb phosphorus in the substrate through roots, but can also absorb and remove phosphorus pollution in water through leaves by synthesizing it to the structural constituents [13]. When it is harvested and transported out of the aquatic ecosystem, nutrients are also exported from the water body to achieve the final removal of phosphorus. The strong adaptability of *M. aquaticum* is reflected in its ability to grow under different concentrations of nitrogen and phosphorus, which is closely related to the molecular mechanism of the plant itself to cope with nutrient stress [14]. Research on the molecular mechanism of *M. aquaticum* to nitrogen stress shows that there are three main molecular regulation pathways in *M. aquaticum* to help it resist ammonium ion stress: (1) increasing the activity of key enzymes that convert harmful ammonium ions into amino acids and proteins; (2) improving the activity of amylase and β-glucosidase to decompose starch and cellulose reserves to obtain more carbon source and energy to resist ammonium ion stress; (3) enhancing the activity of antioxidant enzymes (superoxide dismutase and peroxidase) and the concentration of secondary metabolites (total phenols and flavonoids) to accelerate the removal of excessive reactive oxygen species to avoid secondary damage caused by reactive oxygen species [14,15,16]. However, the molecular mechanism of *M. aquaticum* response to phosphorus stress, especially the gene regulation process related to phosphorus tolerance and metabolism, is still unclear.

The deficiency of macronutrients such as phosphorus is always negative for photosynthesis and growth of plants, and previous studies based on the response of model plants to low phosphorus stress show that plants can activate complex molecular networks to regulate the metabolic pathways of the genome at the transcriptional and post-transcriptional levels to cope effectively with low phosphorus stress, such as inducing the expression of high-affinity phosphorus transfer proteins, and optimizing phosphorus metabolism pathways [5,17,18]. A large number of genes involved in coping with low phosphorus stress have been identified in model plants. For example, in Arabidopsis thaliana, the MYB-CC transporter PHR1 and its homologues PHR1-LIKE1 (PHL1) and PHL2 play a central role in gene transcription and plant metabolic response to different concentrations of phosphorus stress [19,20]. However, these studies focus mainly on the molecular mechanism of low phosphorus stress in model plants (*Arabidopsis thaliana*) or crops (rice, wheat, corn, beans, etc.) in soil and farmland ecosystems, and there are few studies on the response of aquatic plants to high phosphorus stress. 

For the study of plant tolerance to nutrient stress, the traditional method is to explore the function of a single gene by knocking out related genes [21]. Although this method is very effective, it can only study the function of one or several genes at a time, and can only reveal a small part of the rather complex regulatory network of plants from a single aspect, and cannot elucidate the mechanism of plant coping with stress from the genome as an entirety. Therefore, genome-scale studies are crucial to discover plant responses to nutrient stress and metabolic mechanisms, as well as to gain a comprehensive understanding of the regulation of different metabolic pathways in plants and the regulatory networks formed by metabolic pathways [22,23,24]. Our previous studies have shown that *M. aquaticum*’s extremely strong tolerance and efficient assimilation of ammonia nitrogen is regulated by a complex metabolic network in plants at the transcriptome level, which lays a good foundation for us to study the response of *M. aquaticum* to phosphorus stress [14,15]. In this study, to reveal the regulatory network involving multiple metabolic pathways in response to phosphorus stress, *M. aquaticum* was cultivated at different phosphorus concentrations and the growth was measured, and then and the expression of related coding genes and metabolic pathways in the roots and leaves were investigated by high-throughput sequencing technology, and the molecular regulation mechanism of *M. aquaticum* against phosphorus stress were finally elucidated from the perspective of the whole transcriptome.

## 2. Results

### 2.1. Growth Rate and Chlorophyll Pigment Content under Different Phosphorus Concentrations

**Figure 1:**
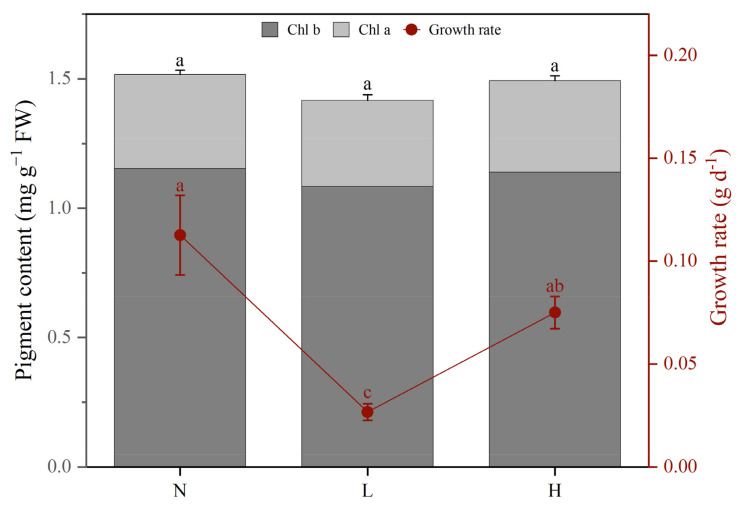
The growth rate and chlorophyll a and b pigment content of *Myriophyllum aquaticum* under different phosphorus concentrations. N, normal phosphorus concentration treatment (0.25 mM); L, low phosphorus concentration treatment (0.02 mM); H, high phosphorus concentration treatment (5 mM). Different lowercase letters above the error bars in the same color mean significant differences, and bars sharing the same letter are not significantly (Turkey’s test).

The growth rate and chlorophyll (Chl a and Chl b) contents of *M. aquaticum* under different phosphorus concentrations were measured and depicted in Figure 1. As shown in the figure, when *M. aquaticum* was exposed to low phosphorus stress, the growth rate was the lowest among the three treatment groups, which significantly decreased by 4.22 times (*p* < 0.01) and 2.81 times (*p* < 0.05) that of the normal phosphorus group and the high phosphorus stress group, respectively. Similarly, low phosphorus stress also exerted a larger impact on root number and root length of *M. aquaticum* than high phosphorus stress (Figure S1). Although high phosphorus stress did not cause significant declines in the *M. aquaticum* growth rate, it led to significant reductions in root length and number. To investigate the influence of different phosphorus concentrations on photosynthesis of *M. aquaticum,* pigment contents of chlorophyll a and b were further analyzed. The results showed that low and high phosphorus stress could not significantly reduce pigment contents of chlorophyll a and b in leaves of *M. aquaticum* (*p* > 0.05), while low phosphorus stress gave rise to greater impacts (Figure 1).

### 2.2. Differentially Expressed Gene Analysis

**Figure 2:**
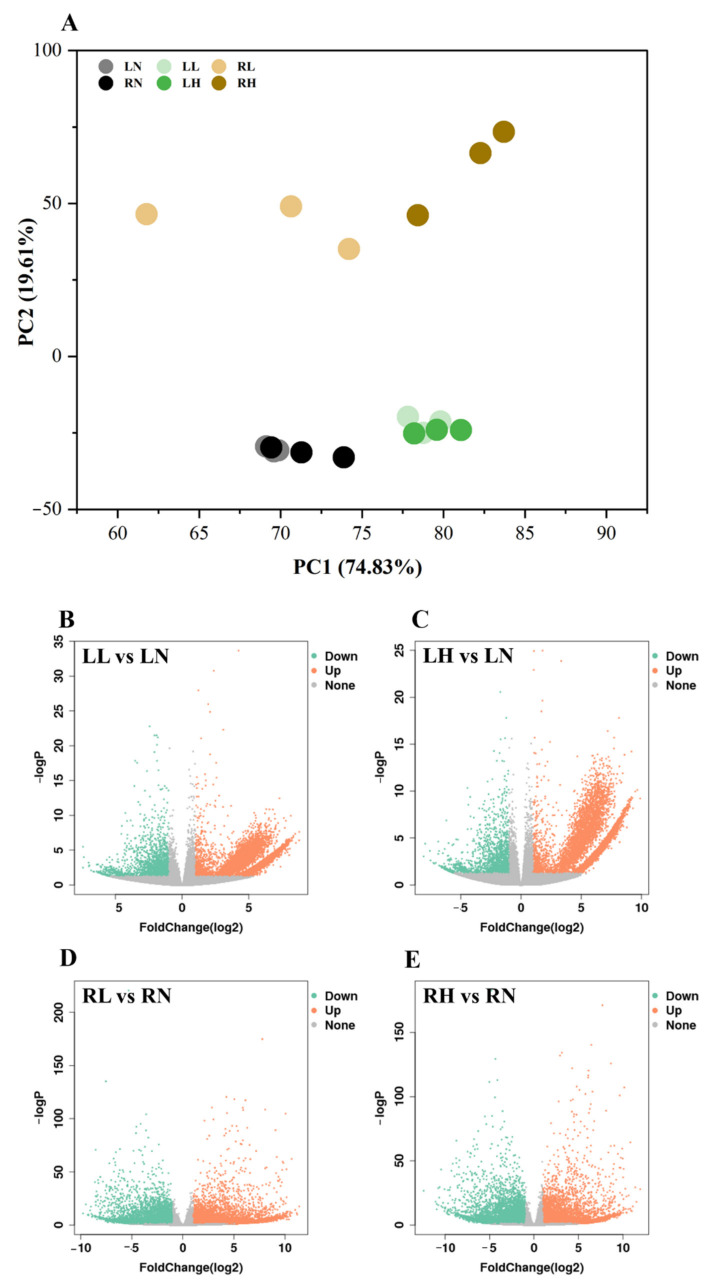
Principal component analysis (PCA) of the gene expression at different concentrations of phosphorus (**A**), and volcano plots of the differentially expressed genes (DEGs) between control and treated *Myriophyllum aquaticum* (**B**–**E**). LN, LL and LH: leaf samples from normal (0.25 mM), low (0.02 mM) and high (5 mM) phosphorus concentration treatment; RN, RL and RH: root samples from normal (0.25 mM), low (0.02 mM) and high (5 mM) phosphorus concentration treatments.

**Figure 3:**
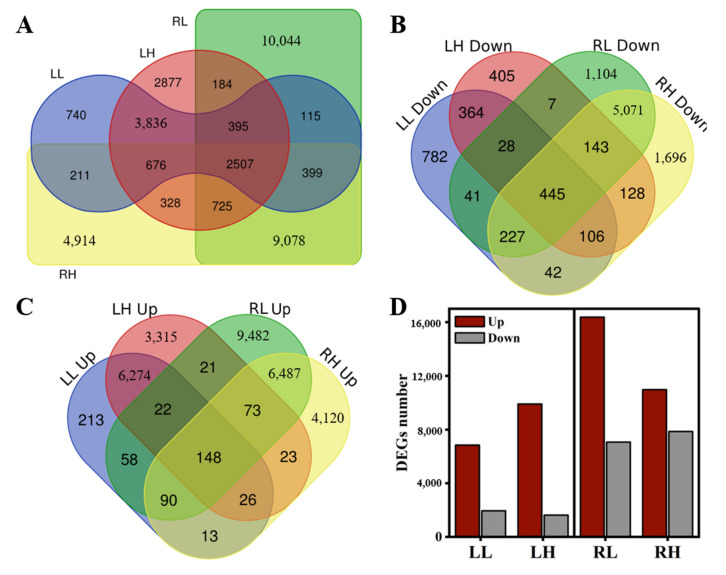
Differentially expressed genes (DEGs) among different groups in *Myriophyllum aquaticum*. Venn diagram of all DEGs, down-regulated and up-regulated genes, respectively (**A**–**C**); Total number of up-regulated and down-regulated DEGs (**D**). LL and RL, leaf and root from low phosphorus concentration treatment; LH and RH, leaf and root from high phosphorus concentration treatment.

To explore the genetic mechanisms regulating the distinct responses of *M. aquaticum* to low and high phosphorus stresses, transcriptome sequencing was performed on 18 samples of roots and leaves tissues from *M. aquaticum* inoculated under different phosphorus concentrations, and 788 million clean reads and 11.82 billion clean bases were obtained after data filtering (Table S1). Compared with raw reads and base, the average clean reads rate of all samples was 85.01%, and the average clean Q30 Bases Rate was 94.54% (Figure S2A), indicating that high sequencing quality was obtained in this study, which could also be indicated by the sequencing quality distribution of each sample (Figure S2B). After assembling the overall clean data with Trinity software, a total of 31,728 unigenes were obtained, and the N50 and N90 of unigene length were 1625 and 557, respectively (Figure S2C). Principal component analysis (PCA) was used to capture the overall variance among the transcript data responses to different phosphorus concentrations. The results showed that the gene expression in samples under low and high phosphorus stresses were separated from samples under normal phosphorus concentration (Figure 2A), indicating striking differences in gene expression profiles. It was worth noticing that samples in groups LL and LH clustered together, while samples in groups RL and RH were separated from each other, especially along the first PC, which explained 74.83% of the variation, and indicated that distinct and substantial transcriptomic responses were caused in roots and leaves tissues of *M. aquaticum* under different phosphorus concentrations. The number of differentially expressed genes (DEGs), whose expression was significantly (*p* < 0.05) altered in roots and leaves tissues of *M. aquaticum* under low and high phosphorus stresses compared with the control group, were further analyzed. In total, the number of up-regulated genes was more than that of down-regulated genes in both low and high phosphorus stress groups (Table S2). Moreover, the volcano plots of the DEGs between the control and treated *M. aquaticum* illustrated that changes of up-regulated genes were larger than those of down-regulated genes (Figure 2B–E). Specifically, there were 2507 common DEGs in all treatments that might be genes related to regulation of phosphorus metabolism (Figure 3A). Furthermore, the numbers of up-regulated DEGs and down-regulated DEGs in roots were both higher than that in leaves, while the down-regulated DEGs shared by roots and leaf tissues were higher than up-regulated DEGs in each group, which were 445 and 148, respectively (Figure 3B–D). Thus, different gene regulation mechanisms may be involved in coping with low phosphorus and high phosphorus stresses for root tissue and leaf tissues of *M. aquaticum*.

### 2.3. Gene Ontology Analysis of DEGs

**Figure 4:**
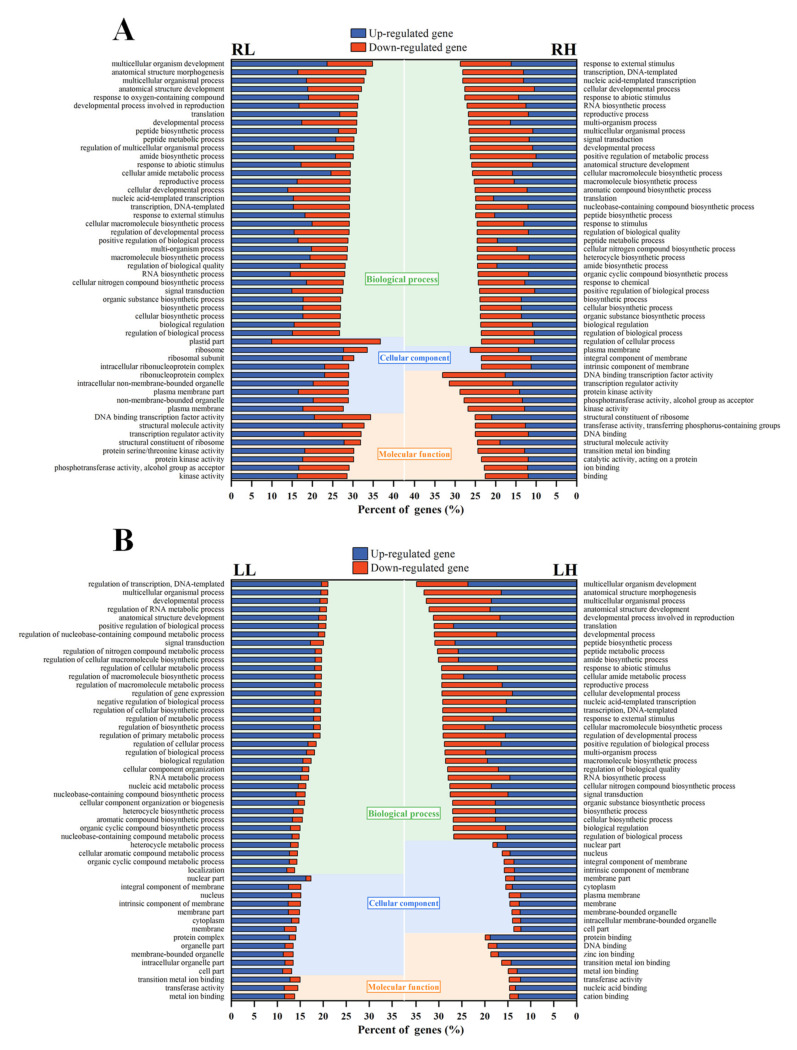
Significantly enriched Gene Ontology (GO) terms (top 50) for differentially expressed genes (DEGs) in roots (**A**) and leaves (**B**) of *Myriophyllum aquaticum.* LL and RL, leaf and root from low phosphorus concentration treatment; LH and RH, leaf and root from high phosphorus concentration treatment.

GO enrichment analysis was further performed on DEGs in root and leaf tissues of *M. aquaticum*, which could provide an overview of the processes most affected by different phosphorus stresses. The distribution of GO terms on the second level according to biological process, molecular function, and cellular component is shown in Figure S3 [25]. Among these three categories, the biological process contained the most enriched GO terms under both low and high phosphorus stresses, mainly involving cellular processes, metabolic processes, single-organism processes, and biological regulation. Under low phosphorus stress, the proportions of up-regulated and down-regulated genes for most GO terms were generally similar in roots and leaves, while the up-regulated genes had a higher proportion for major GO terms under high phosphorus stress. In addition, the degree of up-regulation of individual metabolic processes was obviously greater than the degree of down-regulation, especially in leaves, which was consistent with the result of DEGs analysis. For the cellular component, the variations of GO terms were similar to that in the biological process. For molecular function, a greater differential expression appeared in roots than in leaves under low phosphorus stress, but under high phosphorus stress the differential expression gap between roots and leaves was narrowed or even similar. It is worth noting that, although the types and numbers of GO terms in specific metabolic processes were different in the face of low phosphorus and high phosphorus stress, the overall change trend has a good correlation. The detailed significantly (*p* < 0.05) enriched GO terms on the fourth level are listed in Table S3 and the top 50 enrichment classifications in leaves and roots under different phosphorus stresses are depicted in Figure 4. The commonly involved DEGs in the four groups were all in the biological process, including anatomical structure development (GO:0048856), biological regulation (GO:0065007), developmental process (GO:0032502), positive regulation of biological process (GO:0048518), regulation of biological process (GO:0050789), and signal transduction (GO:0007165). Apart from the common terms enriched in all groups, only one commonly GO term (multicellular organismal process, GO:0032501) was enriched in leaf and root samples under low phosphorus stresses. Under high phosphorus stresses, however, there were 23 additional commonly enriched GO terms in leaf and root samples, which also included DNA binding (GO:0003677), transferase activity (GO:0016740) and transition metal ion binding (GO:0046914) that belong to molecular function, and plasma membrane (GO:0005886), belonging to the cellular component.

### 2.4. KEGG Enrichment Analysis of DEGs

**Figure 5:**
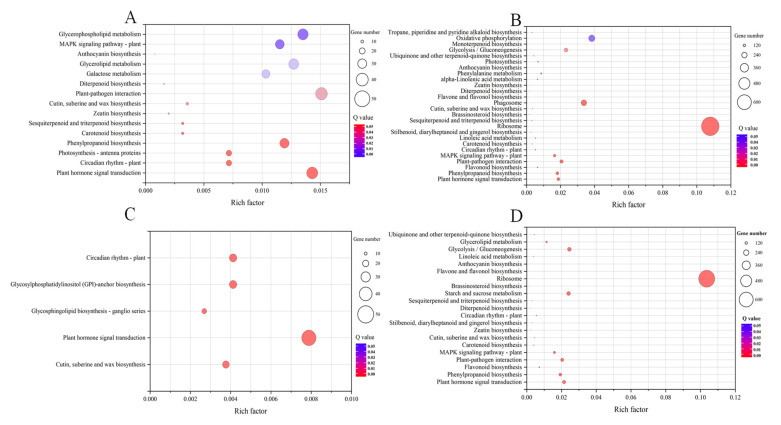
Statistics analysis of Kyoto Encyclopedia of Genes and Genomes (KEGG) enrichment among different groups in *Myriophyllum aquaticum*), leaf and root from low phosphorus concentration treatment (**A**,**B**); leaf and root from high phosphorus concentration treatment (**C**,**D**).

KEGG enrichment analysis was further performed on the DEGs in the root and leaf tissues of *M. aquaticum* and are summarized in Table S4. Figure 5 shows the significantly (*p* < 0.05) enriched pathways in each group. In total, high phosphorus stress led to more significantly (*p* < 0.05) enriched pathways than low phosphorus stress, which were 15, 5, 26, and 21 for LL, RL, LH, and RH, respectively. Pathways of cutin, suberine and wax biosynthesis (ko00073), plant hormone signal transduction (ko04075), and circadian rhythm (ko04710) were commonly enriched in all groups. The translation pathway (Second Category) was one of the most enriched pathways in the root tissues of all groups, and the pathways of energy metabolism, and transport–catabolism were also highly enriched under high phosphorus stress (Table S4). However, the enrichment of these two pathways obviously decreased under low phosphorus stress, indicating that under low phosphorus stress the energy metabolism, transport, and degradation pathways in the root of *M. aquaticum* were severely inhibited. Different phosphorus stresses led to a reverse effect on the glycolysis/gluconeogenesis pathway (ko00010), while commonly enriching these pathways including plant hormone signal transduction pathway (ko04075), biosynthesis of other secondary metabolites pathways, MAPK signaling pathway–plant (ko04016), cutin, suberine and wax biosynthesis (ko00073), plant–pathogen interaction (ko04626), diterpenoid biosynthesis (ko00904), and metabolism of terpenoids and polyketides pathways (Figure 5B,D). Compared with root tissue, the consistency of enrichment pathways was lower in leaf tissue under phosphorus stresses (Figure 5A,C). In response to low phosphorus stress, there are a few more enrichment pathways in the leaf tissues of *M. aquaticum* than that in root, and the enriched cutin, suberine and wax biosynthesis (ko00073), and circadian rhythm (ko04710) were also promoted under high phosphorus stress. Other pathways including plant hormone signal transduction (ko04075), anthocyanin biosynthesis (ko00942), galactose metabolism (ko00052), and photosynthesis—antenna proteins (ko00196) were enriched only under low phosphorus stress. In response to high phosphorus stress, there are fewer enriched pathways in leaf tissue of *M. aquaticum*. Except for the co-enriched pathways with low phosphorus stress, only glycan biosynthesis and metabolism pathways, including the glycosphingolipid biosynthesis-ganglio series pathway (ko00603) and glycosylphosphatidylinositol (GPI)-anchor biosynthesis pathway (ko00563), were enriched under high phosphorus stress. In terms of the total number of activated metabolic pathways, the differential gene enrichment pathways in the root tissue of *M. aquaticum* under high phosphorus stress were less than those enriched under low phosphorus stress.

## 3. Discussion

The water quality of many aquatic environments has worsened due to excessive intake and buildup of trash, particularly phosphorus. In addition to the basic procedures used in wastewater treatment facilities, a variety of innovative treatment options have been extensively created and examined [26,27]. CWs have been widely employed for improved wastewater treatment due to their ecologically beneficial qualities [28]. The macrophytes are one of the most important elements impacting the treatment effects in CWs. As a result, it is preferable if specific macrophytes can survive and effectively clean wastewater with varying levels of phosphorus, especially for these with excess phosphorus concentrations [16]. *M. aquaticum* was recently shown to be one of the few macrophytes capable of not only tolerating wastewater with varying phosphorus contents, but also of efficiently transferring external phosphate into plant tissues. Although *M. aquaticum*’s high phosphorus removal, absorption, and conversion efficiency are well known and frequently published, the underlying processes of *M. aquaticum*’s varied responses to low and high phosphorus stressors remain unknown. This study used transcriptome analysis to look into the genetic basis behind this phenomenon. According to previous research, low phosphorus stress can impact photosynthesis, slow plant growth, and changes morphology, whereas a high phosphorus environment causes plant phosphorus poisoning and decreases agricultural product productivity and quality [13,29,30]. The results of influences of different phosphorus concentrations on *M. aquaticum*’s growth rate in this study indicated that low phosphorus stress had a greater impact on the growth of *M. aquaticum* than high phosphorus stress, which was consist with previous studies showing that *M. aquaticum* preferred nutrient-rich conditions and high phosphorous treatment could significantly increase its branch number and length [31,32,33]. Moreover, previous studies also revealed that shoot porosity was higher in nutrient-rich substrate than in nutrient-low substrate, and the effect of low phosphorous stress was greater in the later period of the growing season of *M. aquaticum* [13,34]. In addition, field observations also suggested that *M. aquaticum* required a high phosphorous environment and could allocate more resources to branch biomass [35]. Meanwhile, more related DEGs could be observed under high phosphorus stress than under low phosphorus stress (Table S2), indicating that *M. aquaticum* could regulate more metabolic pathways to cope with high phosphorus stress and achieve metabolic balance inside the plant [18], which was consistent with the higher growth rate of *M. aquaticum* under high phosphorus conditions. In addition, more DEGs were observed in roots than in leaves of *M. aquaticum,* whether under high phosphorus or low phosphorus stress, which might be due to the direct exposure of plant roots to the high-strength phosphorus solution [4,5]. Similar results were reported with *M. aquaticum* exposure to high-strength nitrogen solutions [15,16]. Specifically, the number of DEGs that were up-regulated was bigger than the down-regulated genes in both the root and leaf under high phosphorus or low phosphorus stress (Figure 3). This result indicates that, quantitatively speaking, *M. aquaticum* may be more dependent on up-regulating the expression of related genes to cope with the external phosphorus stress [2,36]. According to GO enrichment analysis, biological process was the most enriched category in leaves and roots of *M. aquaticum* under both low and high phosphorus stress (Table S3, Figure 4). Many studies have revealed that differentially expressed transcripts in these processes may play an essential role in plant phosphorus stress adaption [2,36]. DEGs involved in photosynthesis, amino acid metabolism (including biosynthesis and degradation of according substances), and carbohydrate metabolism are abundantly expressed under low phosphorus stress in wheat [37]. Under phosphorus shortage, the expression of genes involved in organic acid and amino acid metabolism is significantly altered in oats [38]. In contrast to other plants, different phosphorus stresses had an effect on DEGs involved in anatomical structure development, biological regulation, developmental process, positive regulation of biological process (GO:0048518), regulation of biological process, and signal transduction in *M. aquaticum*.

**Figure 6:**
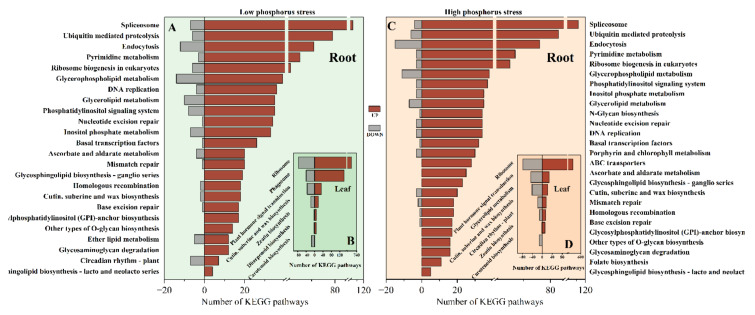
Significantly regulated Kyoto Encyclopedia of Genes and Genomes (KEGG) pathways among different groups in *Myriophyllum aquaticum*, leaf and root from low phosphorus concentration treatment (**A**,**B**); leaf and root from high phosphorus concentration treatment (**C**,**D**).

Photosynthesis provides the necessary energy source for metabolism in green plants and is an important part of their life cycle [39]. The results of changes in chlorophyll a and b of *M. aquaticum* under different phosphorus concentrations indicated that low and high phosphorus stresses did not cause significant decline of their content in leaves, although low phosphorus stress had a greater impact (Figure 1). The effect of different phosphorus conditions on plant photosynthesis could cause different influences on the growth rate of *M. aquaticum* [40]. Stress caused by phosphorus inhibits plant photosynthetic activity and slows their metabolism, thus affecting their growth [41]. A deficiency of phosphorus can negatively affect sugar beet and maize photosynthetic rates, as well as chlorophyll levels. In older and more mature cucumber leaves, phosphorus also causes the veins to lose color [42]. Under low phosphorus stress, DEGs related to photosynthesis were significantly enriched in the leaves of *M. aquaticum*. Other research also shows that there are many DEGs related to photosynthesis in rice under phosphorus starvation [29,43]. Photosynthesis is a crucial aspect of the entire lifecycle of green plants because it supplies the required energy for metabolism [42]. Phosphorus deprivation has been shown to drastically affect the photosynthetic rates and chlorophyll content of sugar beet and maize. [44,45]. Thus, low phosphorus stress may impede *M. aquaticum* photosynthetic activity and cause metabolic slowness, decreasing plant development [46,47]. Under high phosphorus stress, there was no DEG related to pathways of photosynthesis that could be observed. According to the distinct regulatory pattern, *M. aquaticum* demonstrated a higher ability for light capture under high phosphorus stress than it did under low phosphorus stress (Figure 5 and Figure 6).

In addition, under low phosphorus stress, genes that were largely affected and produced regulatory responses were mainly located in roots, especially for genes involved in the following processes: (1) phytohormones biosynthetic and metabolic process, such as auxin and brassinosteroid homeostasis, which is essential for adaptations of plants to biotic and abiotic stresses; (2) cell wall organization and biogenesis; (3) essential substances biosynthetic and metabolic process, including terpenoid, lignin, phenylpropanoid, secondary metabolite, and monocarboxylic acid (Figure 5). It should be noted that genes related to signal transduction, protein phosphorylation, photosynthesis and protein–chromophore linkage in leaves were significantly down-regulated, which could reduce the growth rate while increasing the death rate of plants by cutting down energy requirements [48,49]. Under high phosphorus stress, in addition to roots being affected and producing regulatory responses, leaves were also affected and related genes were differentially expressed, especially by up-regulating the expression. The processes mainly included: (1) cell wall modification; (2) terpenoid biosynthetic process; (3) galacturonan metabolic process; (4) regulation of post-embryonic development; and (5) regulation of seed germination. Meanwhile, genes related to signal transduction, protein phosphorylation, protein–chromophore linkage, and protein localization to membrane were largely down-regulated in roots under high phosphorus stress (Figure 6). Therefore, the root and leaf tissues of *M. aquaticum* actively respond to low/high phosphorus stress by regulating various metabolic processes, and there is a complex regulatory network in the root tissue of the plant, which can better cope with different kinds of abiotic stresses [25,43,50].

**Figure 7:**
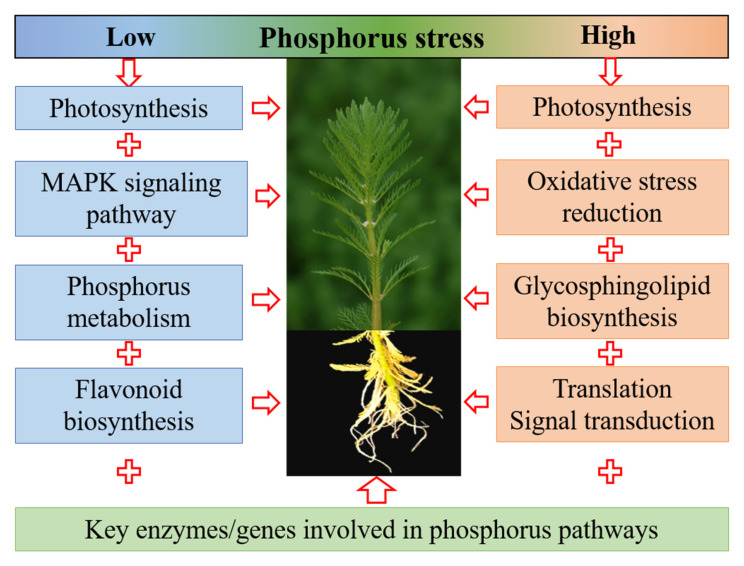
Graphic model of genetic regulatory network in *Myriophyllum aquaticum* responding to different phosphorus stresses.

Enzymes are biocatalysts that control many processes of metabolism, nutrition, and energy conversion. During plant growth and development, many enzymes are activated to resist or adapt to adverse stresses [51,52,53]. The results of KEGG annotation of the expression of genes encoding superoxide dismutase and other antioxidant enzymes showed a larger decline under low phosphorus than high phosphorus stress (Figure 5 and Figure 6), which may lead to more reactive oxygen species accumulation under low phosphorus conditions such as H_2_O_2_ and malondialdehyde [17,21,36]. On the other hand, plant cells interpret phosphorus as a signaling chemical, and high phosphorus concentrations result in lower expression levels of genes involved in phosphorus absorption [54,55]. Under low phosphorus stress, however, key genes engaged in phosphorus metabolism were up-regulated, indicating that these genes contributed to phosphorus absorption (Figure 6) [56,57]. The energy metabolism, and transport–catabolism pathways were highly enriched in the low phosphorus stress group, but their enrichment was significantly reduced in the high phosphorus stress group, indicating that *M. aquaticum*’s energy metabolism, and transport–catabolism pathways were severely inhibited under low phosphorus stress (Figure 6) [44,58,59]. The pathways responsible for phosphorus uptake, translocation, assimilation, utilization, and remobilization were also studied (Table S4). For example, phosphorus transporter gene expression increased in *M. aquaticum* under low phosphorus concentration, showing that these genes contributed to phosphorus absorption under low phosphorus stress. The differences in the enrichment pathways under low phosphorus and high phosphorus stress showed that the complex and diverse metabolic pathways were regulated by *M. aquaticum* in response to different levels of phosphorus stress, enabling it to respond effectively to different levels of phosphorus stress. A graphic model was built to summarize and better understand the probable biological mechanisms of *M. aquaticum* for tolerance to different levels of phosphorus stresses (Figure 7). Under low phosphorus stresses, the genes related to photosynthesis pathways, MAPK signaling pathways, and phosphorus metabolism pathways in leaves, and flavonoid biosynthesis pathway in roots were mainly regulated. Under high phosphorus stresses, the genes related to pathways of photosynthesis, oxidative stress reduction, and glycosphingolipid biosynthesis in leaves, and pathways of translation and signal transduction in roots were mainly regulated. Commonly, key enzymes and genes involved in phosphorus pathways were also functioned to cope with low and high phosphorus stresses. However, the above conclusions drawn merely from statistically significant differential expression of assembled transcripts are relatively preliminary, and should be confirmed by more quantitative methods such as qPCR assay to obtain a more definite conclusion. 

## 4. Materials and Methods

### 4.1. Experimental Design

This study was conducted in a greenhouse under a 12/12 h dark/light photo-period at a stable temperature (26 ± 2 °C). *M. aquaticum* seedlings with a uniform length of ~40 cm and an age of 2 weeks were cultivated in each container (0.5 m × 0.4 m × 0.4 m) with a layer of 10 cm thick fresh quartz sand (average sand diameter of 1–3 mm) evenly spread at the bottom as the attachment medium. A total of 25 L 50% Hoagland culture solution with an identical NH_4_Cl concentration of 0.25 mM and different phosphorus concentrations was loaded in the container, and the group with a phosphorus concentration of 0.25 mM was used as the normal treatment control group; the group with a phosphorus concentration of 0.02 mM was used as the low phosphorus stress group; and the group with a phosphorus concentration of 5 mM was used as the high phosphorus stress group. The phosphorus concentrations of low and high phosphorus stresses were designed based on previous studies [13]. Each group contained three containers as replicate. During the experiment, the culture medium was changed every 2 days to maintain adequate nutrition, and 1 M KOH and HCl solution were used to keep the pH stable at 6.0. After 14 days of culture, 20 mature and healthy *M. aquaticum* plants were randomly selected from each group. The Chl a and Chl b contents in the leaves of *M. aquaticum* were measured with a spectrophotometer (HACH, USA) according to previous report [60]. Briefly, 0.5 g of leaf from each sample was cut out for grinding with 10 mL of acetone at 80%, and acetone was added to a total of 50 mL in each tube. Tubes were stored in the dark at 4 °C for 48 h prior to spectrophotometer measurements. Each sample for pigment determination was filtered, placed in a cuvette and the absorbance measured at 470, 645 and 663 nm with a spectrophotometer. Finally, Chl a and Chl b contents in the sample were calculated by the following Formulas (1) and (2) [61].


(1) Chl a=12.21×A663−2.81×A646

(2) Chl b=20.13×A646−5.03×A663

After measuring their root length and number, chlorophyll pigment content, and growth rate, the plants were thoroughly rinsed three times with sterile distilled water to remove the residual culture solution, and the residual water was dried by suction paper. The root and leaf tissues of the treated plants were cut, grouped for liquid nitrogen quick frozen, and stored in a refrigerator at −80 °C for further use.

### 4.2. RNA Extraction and Library Construction

*M. aquaticum* root and leaf tissue were thoroughly ground in liquid nitrogen, and 300 mg of ground plant tissue samples were taken, and total tissue RNA was extracted and purified with an EASYspin Plus Complex Plant RNA Kit (Aidlab Biotech, Beijing, China). The purity, integrity, and concentration of RNA were determined by Nanodrop 2000 ultraviolet-visible spectrophotometer (Thermo Scientific, Waltham, MA, USA) and Agilent 2100 biological analyzer (Agilent Technologies, Santa Clara, CA, USA), respectively. The NEBNext^®^ Ultra™ RNA Library Prep Kit for Illumina^®^ (#E7530L, Ipswich, MA, USA) was used for sequencing library construction. Three biological replicates of each group were used for RNA-seq analysis.

### 4.3. Transcriptome Sequencing, Assembly and Annotation

The constructed RNA libraries were sequenced using the Hiseq X Ten platform of Illumina by Majorbio Bio-pharm Technology Co., Ltd. (Shanghai, China). The sequencing platform adopted the PE150 paired-end sequencing method. The original sequence data was uploaded to the SRA database of the National Center for Biotechnology Information (NCBI) (PRJNA497710). The raw reads of the original sequencing sequence were quality controlled and filtered using Perl scripts to obtain corresponding clean reads (https://github.com/topics/perl-scripts, accessed on 22 March 2021), which ensured the reliability of the sequencing sequence quality for subsequent analysis. The clean reads were spliced and assembled using TRINITY software (http://trinityrnaseq.github.io/, accessed on 22 March 2021). The assembled transcript sequences and Unigenes were searched and compared with common databases, including Gene Ontology (GO) and Kyoto Encyclopedia of Genes and Genomes (KEGG), and the functional information of sequences in the corresponding databases was annotated.

### 4.4. Differentially Expressed Genes and Enrichment Analysis

The RNA-Seq by Expectation Maximization (RESM) software was used to count the number of sequences of each identified gene in each transcriptome sample, and the Fragments Per Kilobases Million (FPKM) of each unigene in each sample was calculated. For subsequent analysis, unigenes with FPKM ≥ 1 were retained and further adjusted significant difference (FDR) values were calculated for each unigene in each sample using DESeq2 software [62]. When the FDR value of the corresponding unigene was less than 0.05 and the |log2FoldChange| ≥ 1 of each treatment compared with the control sample, the unigene was considered to be a differentially expressed gene (DEG) [63]. After the DEGs were screened out, the enriched metabolic pathways in each treatment group were further analyzed according to their enrichment analysis annotations in the GO and KEGG databases.

### 4.5. Statistical Analysis

One-way analysis of variance with Tukey’s test was applied to identify significant differences between groups and between tissues (*p* < 0.05). A Spearman two-tailed test was used to identify significant correlations (*p* < 0.05) between growth rates in the plant samples. However, there was no significant difference in the growth rate between the high phosphorus stress group and the normal phosphorus group.

## 5. Conclusions

In this paper, on the basis of the comparison of the differences in the growth status of *M. aquaticum* under low phosphorus and high phosphorus stress, the underlying molecular mechanisms and gene regulation networks were further studied. It can be concluded that *M. aquaticum* could cope better with high phosphorus stress compared with low phosphorus stress. Through GO and KEGG enrichment analysis, the metabolic pathways of DEGs enriched in the root and leaf tissues of *M. aquaticum* were deeply investigated, and clearly demonstrated that different gene regulation strategies and metabolic pathways were adopted by *M. aquaticum* to coped with low phosphorus and high phosphorus stress. The complex and diverse gene regulation pathways and networks enabled *M. aquaticum* to cope effectively with different degrees of phosphorus stress. The advantages of *M. aquaticum* in coping with phosphorus stresses may be reflected in the stronger ability on regulation of metabolic pathways including photosynthesis, oxidative stress reduction, phosphorus metabolism, signal transduction, secondary metabolites biosynthesis, and energy metabolism pathway. These findings lay the theoretical groundwork and offer direction for the future application of *M. aquaticum* in CWs for the biological treatment of wastewaters, especially for these with excess phosphorus concentrations. 

## Data Availability

Data is contained within the article or Supplementary Materials.

## Supplementary Materials

The supporting information can be downloaded at: https://www.mdpi.com/article/10.3390/ijms24054874/s1.
