# Supplementary material for: Transcriptomics Insights into Phosphorus Stress Response of Myriophyllum aquaticum
Source: Int J Mol Sci. 2023 Mar 2;24(5):4874. doi: 10.3390/ijms24054874 (PMC10003231; doi:10.3390/ijms24054874)
Supplement: Supplementary file 1 [file ijms-24-04874-s001.zip › Supplementary Figures.pdf]

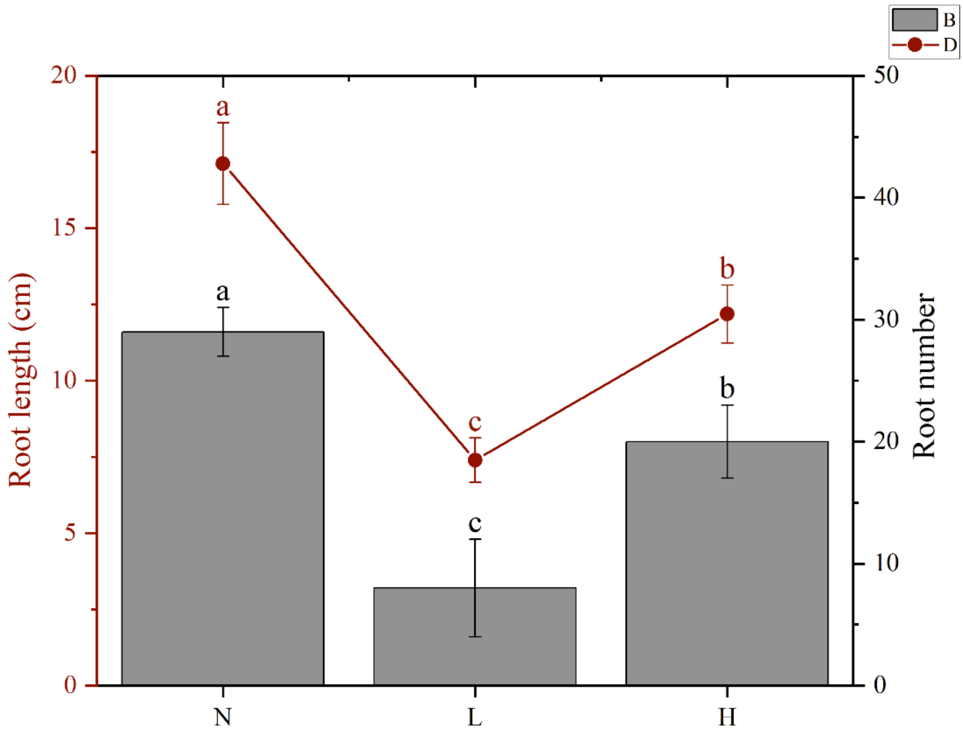

Figure S1. Root number and root length of *Myriophyllum aquaticum* under different phosphorus concentrations. N, normal phosphorus concentration treatment (0.25 mM); L, low phosphorus concentration treatment (0.02 mM); H, high phosphorus concentration treatment (5 mM).

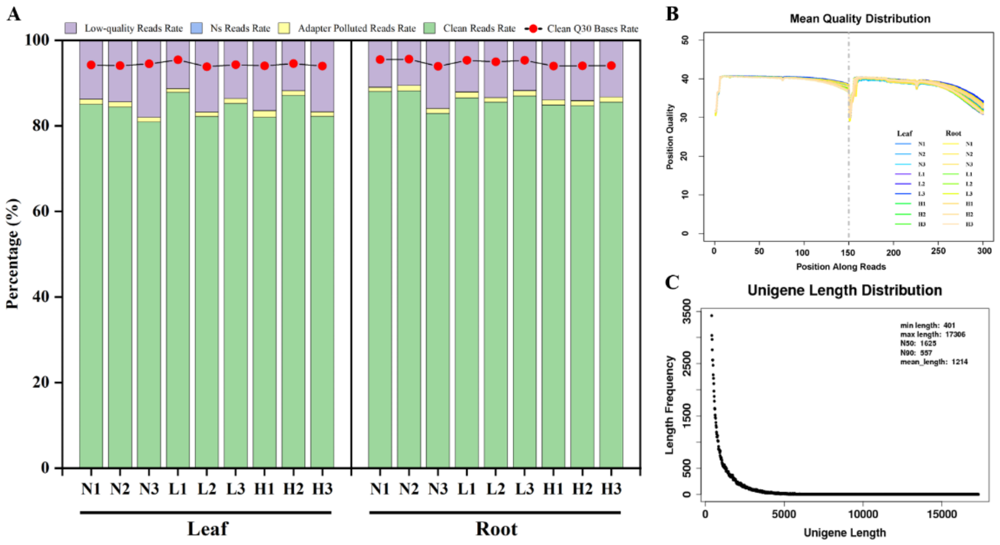

Figure S2. Filter distribution (A), mean quality distribution (B), and unigene length distribution (C) of transcriptome sequencing data; N, normal phosphorus concentration treatment (0.25 mM); L, low phosphorus concentration treatment (0.02 mM); H, high phosphorus concentration treatment (5

mM).

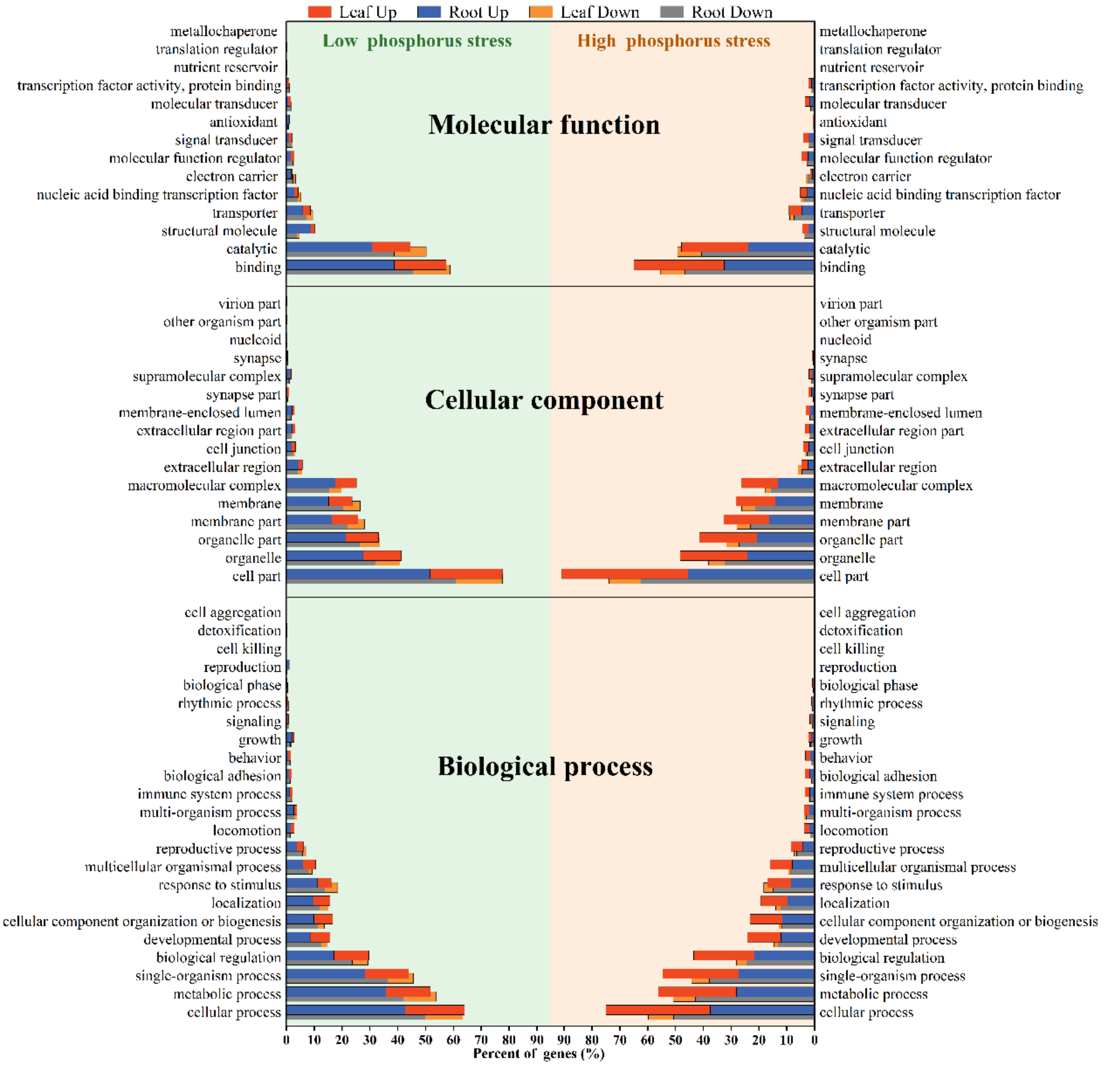

Figure S3. Analysis of Gene Ontology (GO) enrichment for differentially expressed genes (DEGs) among different groups in *Myriophyllum aquaticum*. Leaf Up and Root Up, up-regulated DEGs in leaf and root; Leaf Down and Root Down, down-regulated DEGs in leaf and root.
